# How psychotherapists handle treatment errors – an ethical analysis

**DOI:** 10.1186/1472-6939-14-50

**Published:** 2013-12-09

**Authors:** Irina Medau, Ralf J Jox, Stella Reiter-Theil

**Affiliations:** 1 Clinical Ethics/UPK, Psychiatric Hospitals of the University Basel, IBMB, University of Basel, Schanzenstr. 13, 4056, Basel, Switzerland; 2 History and Theory of Medicine, Institute of Ethics, Ludwig-Maximilians-University Munich, Munich, Germany

## Abstract

**Background:**

Dealing with errors in psychotherapy is challenging, both ethically and practically. There is almost no empirical research on this topic. We aimed (1) to explore psychotherapists’ self-reported ways of dealing with an error made by themselves or by colleagues, and (2) to reconstruct their reasoning according to the two principle-based ethical approaches that are dominant in the ethics discourse of psychotherapy, Beauchamp & Childress (B&C) and Lindsay et al. (L).

**Methods:**

We conducted 30 semi-structured interviews with 30 psychotherapists (physicians and non-physicians) and analysed the transcripts using qualitative content analysis. Answers were deductively categorized according to the two principle-based ethical approaches.

**Results:**

Most psychotherapists reported that they preferred to an disclose error to the patient. They justified this by spontaneous intuitions and common values in psychotherapy, rarely using explicit ethical reasoning. The answers were attributed to the following categories with descending frequency: 1. Respect for patient autonomy (B&C; L), 2. Non-maleficence (B&C) and Responsibility (L), 3. Integrity (L), 4. Competence (L) and Beneficence (B&C).

**Conclusions:**

Psychotherapists need specific ethical and communication training to complement and articulate their moral intuitions as a support when disclosing their errors to the patients. Principle-based ethical approaches seem to be useful for clarifying the reasons for disclosure. Further research should help to identify the most effective and acceptable ways of error disclosure in psychotherapy.

## Background

In 1979, the American sociologist Charles Bosk conducted the first empirical research on medical errors and published a seminal book about his insights into forms of error and their management in surgery [1]. However, it was not until the year 2000 when the Institute of Medicine’s report “To Err is Human: Building a Safer Health System” spurred the modern patient safety movement [2]. While errors in medicine have since been intensively studied and discussed, errors in psychotherapy have rarely been addressed [3]. One possible reason for this is that the very concept of error in psychotherapy is vaguely defined. One element of an error is the breach of a widely accepted standard of care. These standards, however, are barely established in psychotherapy, which may be due to the lack of evidence-based guidelines, the methodological difficulty of comparative research and the diversity of psychotherapeutic schools.

Based on our own interview study with psychotherapists (PTs) we categorized psychotherapeutic errors into technical, judgemental, normative and system errors – similar to how Charles Bosk categorized medical errors [1,4]. In psychotherapy, technical errors may concern the diagnostic work-up or the procedure of a specific behavioural therapy. Judgemental errors refer to the choice of a wrong therapeutic method or the misjudgement of the client-therapist relationship. A normative error occurs, e.g. when confidentiality is breached. System errors describe organisational failures (e.g. lack of time or failure to initiate a follow-up therapy). This framework represents the first part of our project and was used as a background for the following study.

An ethically salient question is whether errors should be disclosed to the patient. The shift to a more patient-centred approach has refocused attention on patient autonomy. Patients are generally taking a more and more active role in treatment planning, decision making and evaluation [5]. In medical ethics, the patient’s “right to truth” has been firmly established and grounded mainly in the respect for patient autonomy, the harm of dishonesty, the necessity of informed consent for follow-up care and the importance of trust for any health care delivery [6]. Yet, the obligation to tell the truth may collide with the duty to minimize harm if truth telling itself will almost certainly lead to severe damage and undermine the treatment effect. This may particularly be the case in mentally ill patients [7]. For these specific situations a “therapeutic privilege” to hide the truth has been discussed [8]. This position however is now largely discredited as a form of unjustified paternalism. Even if medical information about diagnosis or prognosis may be delayed in exceptional circumstances, the therapeutic privilege does not apply to the error disclosure because the patient’s right to truth is held to be even stronger if he has been harmed by the professional [9,10].

The same arguments for error disclosure apply to psychotherapy, yet PTs may fear a disruption of the therapeutic relationship, a breakdown of the therapy or even serious harm to the patient like suicide. The ethical analysis and conclusion about the question whether to disclose errors in psychotherapy or not critically depends on the underlying ethical theory. In deontological theories, especially in Kantian ethics, the duty of truthfulness is very powerful, and lying will violate Kant’s Categorical Imperative [11]. Consequentialism, however, evaluates actions only according to their likely consequences, classically whether they maximize happiness in the world. In this view, lies and deception may be ethically legitimate, and the question of error disclosure will depend on the anticipated consequences in a given case. Empirical research on medical errors supports open disclosure [5], but studies also show that the consequences depend on the way the error is communicated [12-14].

The second key topic discussed here is the management of errors made by colleagues. Professional codes of ethics for psychotherapists (e.g. of the British Association for Counselling and Psychotherapy or the Professional Association of German Psychologists BDP) do not refer to errors. However, they do emphasise the duty to exercise objectivity towards colleagues. Objectivity and collegiality seem to play an important role when handling errors, and psychotherapists are particularly unsure of how to manage such situations [15].

The moral judgement and practical management of one’s own errors and those of colleagues is influenced by the ethical culture of the profession. Which one can we expect of psychotherapy? In professional psychotherapeutic guidelines and codes of conduct, the management of errors is not explicitly mentioned, yet the Principles of Biomedical Ethics formulated by the Beauchamp & Childress (B&C) [16] have been welcomed as a guide in psychotherapy [17]. They are part of the curricula for trainee psychotherapists and referred to in different professional codes. The principlist approach is based on common-sense morality and consists of four duties: respect for autonomy, non-maleficience, beneficence and justice. Lindsay et al. (L) formulated a modified set of ethical principles and specifically aim to account for the ethical requirements that psychologists (and other practitioners) face in psychotherapy in Europe: respect, competence, responsibility and integrity [18,19]. Each of these principles is reflected in a statement of relevant values and a set of specific standards. Respect is divided into general respect, privacy, informed consent and confidentiality. The principle of competence describes ethical awareness and knowledge. Knowing the limits of one’s own competence and taking advanced training are also subsumed under this principle. The principle of integrity describes recognition of professional limitations through self-reflection, honesty and accuracy. Also included are describing one’s own qualification accurately and the obligation to comment on colleagues’ actions if they seem wrong. The principle of responsibility calls for the best possible treatment and the avoidance of harm.

Whilst both approaches readily overlap in certain areas, they diverge in others. In contrast to the medical case studies in the work of B&C, L use examples from psychotherapy. The Lindsay approach has been adopted by the Meta-Code of Ethics of the European Federation of Professional Psychological Associations [20]. Moreover, the Code of Ethics and Conduct of the British Psychological Society (BPS) and the German “Bund Deutscher Psychologinnen und Psychologen” introduce the Lindsay principles [21].

Medical studies showing beneficial consequences of the honest disclosure of an error encourages the development of ethically sound error management standards. As a first step in this direction, we conducted an interview-based study with psychotherapists and aimed to explore:

1. PTs’ preferred ways of dealing with an error made by themselves and/or by a colleague;

2. PTs’ reasons for their preferred course of action in the light of the two existing principle-based ethical approaches (B&C and L);

3. Ethically sound recommendations for handling errors in psychotherapy through discussion.

## Methods

### Setting

We chose a qualitative design due to the exploratory nature of our first two research questions and the need for in-depth insights into subjective perspectives [22]. Data were collected using semi-structured interviews with PTs working in the greater area of Berlin, Germany. Ethical approval was obtained from the ethics committee of the local PT trade association.

### Sample

Using a list of accredited supervisors from the national psychotherapist association, 35 PTs, (physicians/psychiatrists or psychologists working as PTs) were contacted for interviews. Snowballing enabled further contacts. Data collection was stopped with theoretical saturation, i.e. when interviews were not adding any further insights. Stratified cluster sampling ensured that the most common types of psychotherapy (psychoanalysis, cognitive-behavioural and client-centred therapy) were included. Five PTs declined to be interviewed due to time pressures. The final sample size was 30. The response rate was 85%.

### Data collection

Based on literature searches (PubMed, Medline, PsychInfo, ETHMED, Bioethicsline) and two co-authors’ own experiences as practicing PTs, a problem-centred interview guide was developed. After a revision process by five experts in qualitative research, psychotherapy, law and forensic medicine, it was tested in a preliminary study with psychotherapy trainees. Three main topics surfaced: 1. Categorisation of errors, 2. Management of errors, and, 3. Ethical approach towards error disclosure. This paper reports on the latter of the three [4,23].

All interviews were conducted by the same interviewer (first author, MSc Psych, trained cognitive-behavioural therapist), took place in the psychotherapists’ offices and lasted approximately one hour. Prior to the interview the participants signed a consent form and were assured anonymity. In order to focus the PTs on the topic, an operationalized definition of error from the German management handbook for PTs was read out. It translates: “An error is defined as *inappropriate conduct during therapy or an incorrect diagnosis or a false indication, contrary to currently accepted guidelines or standards. An error can also violate basic rules of therapeutic behaviour towards the patient.”*[24]. This was followed by the interview questions using a semi-structured interview approach. The audio-recordings were transcribed verbatim and transcripts were anonymized.

### Analysis and categories

For the analysis of the transcripts we used qualitative content analysis according to Mayring [25]. This is a commonly applied framework approach of systematic, rule-guided text analysis and tries to preserve some methodological strengths of quantitative content analysis extending them to a concept of qualitative procedure. Data were categorized according to a mixed inductive and deductive coding scheme. The inductive coding focused on the PT’s disclosure and management of error. We categorized participants’ answers according to the two sets of principles of B&C and L. This involved the reconstruction and clarification of the observed empirical phenomena of interest by articulating implicit assumptions and underlying meaningful components in explicit ethical terms. This is a type of qualitative approach used in medical ethics research [26]. To enhance reliability, a second coder (psychologist trained in qualitative analysis) analysed 40% of the text material that was randomly selected and matched it with the first coder. The inter-coder reliability was 0.93. Coding was supported by the software MAXqda 2001 (VERBI GmbH Berlin).

## Results

Among the 30 PTs taking part in the interviews, 21 were psychologists, seven physicians (psychiatrists) and two social workers (see Table 1). The distribution of gender (female/male: 2/1) and profession (psychologists/psychiatrists: 3/1) reflects the distribution in the German PT population [27]. The three main psychotherapy approaches were represented: 11 psychoanalysts, 16 cognitive-behavioural therapists, and 3 client-centred therapists. Furthermore, 18 were also qualified as supervisors of other therapists.

**Table 1:** Demographic information of interviewed PTs

	**Characteristic**	**Data**
Gender (n)	Male	9
	Female	21
Age (years)	Mean/Median	45/46
	SD	11
	Range	28-70
Professional setting (n)	Outpatient care	20
	Inpatient care	10
Practical experience (years)	Mean/Median	18/18
	SD	12
	Range	3-40

Legend: *SD* standard deviation.

**Figure 1:**
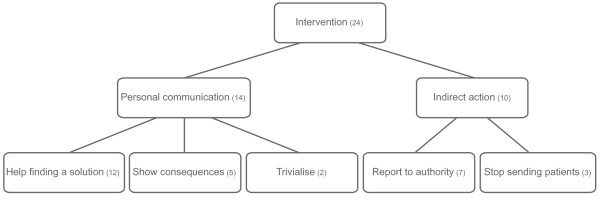
**Actions as responses to errors of colleagues.** Legend: (n) of participants referring to this action, more than one answer was possible.

The majority of PTs reported a practice of disclosing their errors (n = 26/30). They justified this mainly by saying that this improves the therapeutic relationship and allows role model learning for the patient. Their main reason for not disclosing errors was the fear that the patient may cease therapy, leading to harm and a reduced chance of recovery from the mental illness. With respect to errors made by colleagues, the majority of therapists reported that they would take action (see Figure 1).

**Figure 2:**
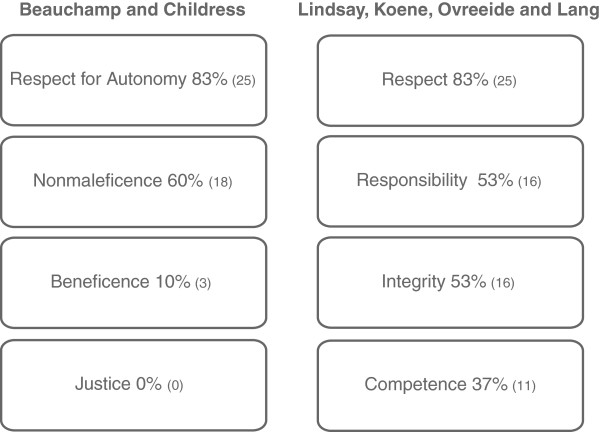
**Reported reasons for/against error disclosure categorized according to the principles of Beauchamp and Childress and Lindsay et al.** Legend: Percentage and (n) of participants referring to that principle. For disclosure more than one ethical justification was given. Non-disclosure was only mentioned with reference to Nonmaleficience or Responsibility (n = 4).

In this study PTs mainly reported their own experiences to explain their error management. When asked about theoretical, especially ethical grounds for their actions, they described relying on intuition or personal values. Directly asked about their ethical training and knowledge, they often stated uncertain or rudimentary training. The majority mentioned a need for further ethical training. In a second step the answers concerning ethical considerations about error management were categorized according to the principles of Beauchamp and Childress and Lindsay et al. (Figure 2).

Most commonly the PTs referred to the category respect (for autonomy) (L, B&C). Several PTs stated that they would like to be informed about errors themselves. Furthermore they referred to integrity – e.g. by characterising error disclosure by the values of honesty and openness towards the patient. Enabling therapy to continue by disclosing an error was categorized under the principles of beneficence (B&C) and competence (L)*.* Many PTs considered error disclosure to be a potential risk of harm, which falls under the principles of non-maleficence (B&C) or responsibility (L). Nearly all of the interviewed PTs emphasized their positive experiences in disclosing errors to their patients. Most PTs said that disclosing an error also meant apologising.

### Examples^a^

“I had rather positive experiences (with disclosure)… Contrary to previous fears, disclosure did not lead to a breach of trust, instead I experienced the opposite…” (T7)^b^.

In one example a psychoanalyst reports about an error concerning counter transference; the redirection of one’s own feelings on the patient, a typical psychoanalytic technique. In this example it can be seen that the psychoanalyst believes there is a difference between psychotherapeutic schools in their attitudes towards disclosing errors:

“I can tell you an example, when I treated a patient wrongly. This patient made me furious and I reacted in an unfriendly manner, which I felt was an error (…). In this case I couldn’t talk about the situation immediately… I could only talk about the situation one year later. This is a huge difference to CBT [cognitive-behavioural therapy] therapists (…) where this could have been dealt with immediately. I see things and keep them in mind, but decide when and how to talk about them. (T3, psychoanalyst, psychologist).”

In another example, a psychoanalyst reported disclosing errors immediately and directly when talking about a judgemental error. In this example there is no difference between psychotherapeutic schools in terms of when an error is disclosed.

“Sometimes you tend to overstrain a patient. When I notice this during the session, I will disclose the error immediately. And when I notice it later, I will start the next session by saying that I have to tell you something important…. I will take responsibility for the error and apologize…” (T13, psychoanalyst, psychologist)

For more examples see Table 2.

**Table 2:** Example statements on the reaction to one’s own errors and ethical reconstruction

**Examples categorized according to the principles of Beauchamp and Childress**	**Examples categorized according to the principles of Lindsay et al.**
**Respect for Autonomy**	**Respect**^**d**^
“The autonomy of the patient is relevant: Can I tell my patient about the error? What do I risk? Things like termination of therapy… But I can’t live with “not telling” the patient. It’s always about treating others, as you would like to be treated yourself (…). This would be a short form of my ethical belief: I do not want to be patronized or stigmatized…I want to be treated autonomously” (T4, CBT, psychologist)^c^	“Respect in treating patients, e.g. not judging them, their decisions – especially when disclosing an error.” (T23, psychoanalyst, psychiatrist)
**Non-maleficence**	**Responsibility**
“I would be inclined to tell the patient (about the error)… But there are a few patients, particularly in psychiatry, when I prefer to not talk about an error. For example when a patient suffers from delusions…” (T12, CBT, psychiatrist)	“I will take responsibility for the error, apologize and express my regret. This has always raised a lot of positive reactions…” (T7, client-centred therapist, psychologist)
**Beneficence**	**(Professional) Competence**
“The patient can benefit from me disclosing an error. They know they can trust me and it is not their fault if therapy does not work. A common error in my opinion is blaming patients for therapy failures.” (T22, CBT, psychologist)	“A common error ([is]) when therapists don’t know their limits. When therapists treat patients without having the necessary background knowledge or professional training…” (T15, psychoanalyst, psychologist)
**Justice**	**Integrity**^**e**^
This category was not coded	“Integrity – this is to not misuse one’s position of power… Integrity also means transparency. Taking this into account, I think there is a duty of disclosure…” (T4, CBT, psychiatrist)

### Participants’ statements on managing the errors of colleagues

“Consequences of errors: not referring patients [to that therapist] anymore and spreading that news or even reporting the therapist to the professional association.” (T3, psychoanalyst, psychologist).

“I would want to protect the patient. As a consequence of an error I talk directly to colleagues, and if necessary, I would certainly deal with the error at a higher level. An error should have consequences. However, first I would talk to the colleague personally.” (T6, CBT, psychiatrist).

The category of non-maleficence was used when PTs described how they prevented harm resulting from errors of colleagues. Both quotes could also be assigned to the Lindsay principle of responsibility.

### Application of ethical principles

The partial overlap of the two principle-based approaches (B&C, L) is mirrored by the results. Evidently, the same PTs’ statements could be categorized both under the principle of respect for autonomy (B&C) and under the principle of respect according to L. Responsibility (L) seems to overlap with the more general principle of non-maleficence (B&C) [16], both in relation to protecting the patient from harm. Similarly, the principle of competence (L) reflects the intention to benefit the patient (beneficence; B&C). The principle of integrity (L) was categorized several times; it functions as an additional category for analysing the PTs’ reasoning when managing errors. No reference could be found in the interview transcripts to fairness (B&C). In conclusion, the data provide a limited fit to the approach of B&C; the matching is improved by the supplementation of the more specific principles of L, especially regarding the additional principle of the psychotherapist’s integrity.

## Discussion

### Error disclosure

According to our PTs, disclosing errors mainly results in a positive outcome. This lends weight to the importance of the duty to disclose errors and supports findings within medical literature [13]. The warning that concealment of an error can lead to irreversible disturbances in the psychotherapeutic relationship, rendering further treatment complicated or impossible [28], is consistent with the reported experiences of the interviewed psychotherapists. Conversely, other authors suggested that disclosing errors could be distressing for the patient and that it may not be right in every clinical context [28,29] – a fear shared by our participants, even though (medical) studies do not support this finding.

Various ethical obligations such as respecting patient autonomy, enhancing their wellbeing and preventing harm coexist. The majority of the reasons the PTs gave to justify their handling of errors falls into one of two categories from the two principle-based ethical approaches [16]. Most commonly, PTs referred to respect for autonomy (B&C)/respect (L) [16,18]. This corresponds with a deontological approach represented by ethical guidelines [30] and the law. Some PTs argued that they would like to be informed about errors themselves referring to the “golden rule” or ethics of reciprocity in favour of a pro-disclosure approach.

The observation that PTs highly value the notion of respect for the patient is not surprising and this idea adopts the medical trend towards patient-centred treatment. Moreover, the idea of patient autonomy has been emphasized in psychotherapy from its conception and can be seen expressed in the movement of psycho-education [28,31]. Another important justification given by the PTs who were pro error disclosure was integrity. The principle of integrity was often mentioned when talking about the asymmetry of the therapeutic relationship or referring to values of honesty and openness towards the patient.

Disclosing a treatment error can allay patients’ fears of being responsible for a failure of the therapy. This reflects both, the principles of beneficence (B&C) and competence (L)*.* Disclosure also provides the patient with the assurance and hope that further decisions will be made in his or her best interest [32]. However, beneficence was addressed explicitly by very few PTs, which is an unexpected finding in the light of the caring motivation inherent in the psychotherapeutic profession. Given that beneficence and non-maleficence are both addressing patient interest and wellbeing, but from two opposing perspectives, this observation could be explained by the PTs focusing more on preventing the harm that errors mean for the patient.

In general, the duties of beneficence and non-maleficence mean evaluating the benefit-burden ratio throughout the therapy process, but especially at its beginning. This should be discussed with the patient as part of obtaining informed consent [33]. Our PTs voiced similar considerations for the phase after disclosure of a treatment error had taken place.

One key component of effective disclosure, as found in the medical error literature [34], is apologising for errors. This became evident in the answers of the PTs as well. Another important component is to enable steps to reverse the error or at least mitigate negative consequences for the patient. Accordingly, PTs in this study emphasized their endeavour to make the best out of an error. For example, an error could be used to adjust the treatment approach. Reversing an error may help to maintain a positive relationship with the patient [35] and this seemed to be highly important to our interviewees. This may explain the frequent statements referring to non-maleficence (B&C) or responsibility (L). The concept of error reversibility is central for the conviction that error disclosure is helpful. The principle of non-maleficence (B&C) has been prevalent in medicine since the outset. Psychotherapy has taken longer to adopting this notion; however, the PTs also considered that error disclosure could lead to the patient discontinuing the therapy with possible harmful effects. Therefore, error disclosure may be discussed under the notion of “therapeutic privilege” [8]. Similar to the interviewed PT who advocated non-disclosure for patients with delusions, we can find a few examples in the literature where patients with poor insight, especially in cases of psychotic disease, are treated in this way [36]. However, the ethical justification of a “therapeutic privilege” is markedly weaker in error disclosure than in handling the information about diagnosis and prognosis in general. This is because a patient who has been wronged has a special right to the truth about this wrongdoing.

On closer scrutiny, disclosure of errors in psychotherapy seems to be influenced by further factors – again comparable to the medical literature [37]. For example, the PTs first wanted to know the patient’s ability to cope with that information in order to make a decision whether to tell the patient about an error. Thus, an individual assessment considering the outcome of the disclosure and its benefit-harm balance was preferred. This reflects a consequentialist approach, aiming to deliver good and desirable outcomes. On the whole, we observe a mix of deontological and consequentialist reasoning in the PTs’ decision making.

Interestingly, the interview data hardly revealed any differences in error management between therapists of different schools. Even though the therapists themselves expected a difference, they rather unanimously favoured disclosing errors. As exemplified in the quote above, psychoanalyst PTs work with rules of abstinence and (counter)-transference [38]. Disclosure at the wrong point of time (i.e. immediate) may go against the requirements of these concepts asking the PT to first reflect before taking any action including error disclosure or apology. However, when not talking about those “special cases”, our psychoanalyst PTs still report disclosure of errors and apologizing for them. This topic warrants further research. It is possible that the PTs who took part in our study, were particularly interested and open-minded regarding the topic of error.

One main finding of this study is that the interviewed PTs emphasized their positive experiences in disclosing errors to patients. This supports Bienenstein and Rother [29] who conclude that the success of therapy depends less on the fact of whether an error was made or not, but on how the error was communicated and managed.

### Responding to errors made by colleagues

In the analysis of the reported management of errors made by colleagues, the principles of non-maleficence (B&C) and responsibility (L) were found to be paramount. Similar to a medical study [39], the PTs argued in favour of the discussion of errors with colleagues, especially targeted at collegial support. In medicine, studies found that this approach enables peer learning by seeing each other as role models [39]. A similar range of considerations was found in our study.

The frequent reference to a character of integrity may reflect the significance of virtue ethics [39-41] for individual orientation as a psychotherapist. It suggests that human qualities play an important role for the profession and personal virtues of (famous) psychotherapists might offer a general ethical orientation. However, this alone would probably fail to provide a precise direction of concrete action, especially when facing ethical dilemma. Therefore, different authors suggested that the approach of virtue ethics should only be used to compliment ethical standards [42]. So far, virtue ethics has rarely been part of professional guidelines or ethics codes. Furthering this approach could be fruitful, especially in relation to education or supervision.

Some of the interviewees reported experiences with colleagues who had refused to reflect upon their errors. Even though they felt the need to intervene in order to protect the patient, they appeared to be reluctant to question their colleagues’ skills. Collegial loyalty seems to cause a conflict with the principle of responsibility [18], although the duty to disclose misconduct or incompetence is ethically well-founded [31]. This result is compatible with more general studies showing a significant difference between “should” and “would do”. Research indicates that PTs “*would”* do less than they knew they “*should”* when confronted with unprofessional or unethical conduct on the part of colleagues [43]. A possible approach to solving this conflict mentioned in the literature was suggested in our interviews as well: initiating a personal contact with a colleague (or talking about the error in a trustworthy supervision environment) before taking any further steps. This would also comply with recommendations by various codes of ethics (e.g. American Psychological Association APA, BPS, BDP) [20]. Different ethical guidelines for psychologists require that psychologists who observe colleagues engaging in moral violations should try to address and resolve these informally in the first instance. Further action should only be taken, if this proves fruitless. However, the threshold to take further steps is high and the PTs in our study tend to avoid it. Besides professional loyalty, this may also be explained by wanting to avoid personal costs, fearing negative repercussions or having insufficient information about ethical guidelines [15]. Research shows that professionals are most in danger of misconduct when those around them do not hold them accountable [15,28]. This means that PTs seem to be responsible for policing themselves and thus need a good understanding of ethical standards [15,28]. Supervision, Balint groups and Morbidity-and-Mortality Conferences (medicine) can be used as model platforms for discussing the topic of errors in psychotherapy; even ethics consultation services could be asked for help, where established. Taking responsibility for each other within teams or institutions should be encouraged together with promoting transparency when it comes to errors. Additionally, options for therapists and/or patients like conciliation committees to mediate cases of error should increasingly be introduced [28]. As one example the American Psychological Association (APA) offers such a committee [44].

### Limitations of the study and outlook

Qualitative research methods have certain limitations. One disadvantage of the current design is that the data are not statistically representative. In addition, although two coders and an interdisciplinary panel were continuously involved in critical review, the coding process itself is open to subjectivity. Issues of validity arise because reported PT behaviour may not directly correlate with their real practice. Observational studies of the disclosure process (if possible) might afford higher validity, but such methods are not without their limitations. As a follow-up to this explorative research, studies investigating the patient’s experience of the consequences of honest error disclosure may prove rewarding. Additionally, research exploring how ethical training could help PTs to successfully deal with errors appears promising.

## Conclusions

Clear ethical recommendations for the disclosure of psychotherapeutic errors have so far been lacking, especially due to the paucity of treatment standards and the little empirical research that is currently available in psychotherapy. Our findings support the views of different psychotherapeutic authors, that adequately disclosing an error has positive effects on the patient.

On the whole, the emphasis placed on respect for patient autonomy on the one hand and on preventing harm to patients on the other seems to give an ethically robust reference point. However, this is not a simple remedy for finding solutions. Clearly, the best ethical way forward for error disclosure seems to be considering the type of error made in combination with the personality (stability) of the patient. This implies tailoring each disclosure to the individual’s situation for maximum effectiveness. This assumes that cases might be handled differently based on reasons such as the capacity of a patient to benefit from certain information. It also implies that professionals need to consider how to make information available to the patient in an empathic manner. This recommendation is in accordance with ethical PT behaviour according to several ethical manuals (e.g. American Psychological Association APA, BPS, BDP). It also corresponds to professional medical guidelines regarding the disclosure of bad news.

Further research is needed to investigate how such individual solutions could best be adjusted according to the situation and the individual patient and what the most effective and acceptable ways of error disclosure might be. It appears helpful to not only rely on intuitive, subjective feelings when seeking guidance, but to engage in explicit ethical reflection professionally. Training PTs in error management and ethics therefore is important.

When handling errors made by colleagues, insecurity and unease to take further steps often prevails – especially in severe cases. Thus, efforts should be taken to offer clearer guidance to PTs in such situations.

Of note were the rather unconfident responses of many PTs who could not rely on conceptual knowledge in relation to ethical principles and desired in-depth ethics training. An unanswered question remains as to whether national approaches/guidelines or cross-border activities will yield better support. The exchange of ideas and experiences is needed, and international debate could enrich such exchange.

## Endnotes

^a^Translated literally from German by first author.

^b^Interview Identification Number.

^c^Interview Identification Number.

^d^The principle of respect by Lindsay et al. subsumes several forms of respect (including e.g. respect for autonomy). Therefore the quotes for autonomy categorized to the Beauchamp and Childress approach have been categorized here as well.

^e^Besides quotes directly referring to integrity, the principle of Integrity was categorized when PTs referred to necessary positive characteristics of PTs (e.g. honesty).

## Competing interests

The authors declare that they have no competing interests.

## Authors’ contributions

IM contributed to the conception and design of the study, carried out the interviews and interview analysis and drafted the manuscript. RJJ contributed to the analysis and interpretation of data and revised the manuscript. SRT contributed to the conception and design of the study, supervised the interview study and interview analysis and revised the manuscript. All authors read and approved the final manuscript.

## Pre-publication history

The pre-publication history for this paper can be accessed here:

http://www.biomedcentral.com/1472-6939/14/50/prepub
